# Bone ongrowth and mechanical fixation of implants in cortical and cancellous bone

**DOI:** 10.1186/s13018-020-01696-5

**Published:** 2020-05-14

**Authors:** William Robert Walsh, Matthew Henry Pelletier, Nicky Bertollo, Vedran Lovric, Tian Wang, Per Morberg, William Chase Harington Parr, Dario Bergadano

**Affiliations:** 1 Surgical and Orthopaedic Research Laboratories (SORL), Prince of Wales Clinical School, UNSW Sydney, Kensington, Australia; 2 Department of Surgical and Perioperative Sciences, Umea University, Umea, Sweden; 3 Medacta International, Lugano, Switzerland

**Keywords:** Bone ongrowth, Mechanical properties, Plasma sprayed, Hydroxyapatite, Animal model, Histology

## Abstract

**Background:**

What is the right surface for an implant to achieve biological fixation? Surface technologies can play important roles in encouraging interactions between the implant surface and the host bone to achieve osseointegration. Preclinical animal models provide important insight into in vivo performance related to bone ongrowth and implant fixation.

**Methods:**

A large animal model was used to compare the in vivo response of HA and plasma-sprayed titanium coatings in a well-reported adult ovine model to evaluate bone ongrowth in terms of mechanical properties in cortical sites, and histology and histomorphometry in cortical and cancellous sites at 4 and 12 weeks.

**Results:**

Titanium plasma-sprayed surfaces outperformed the HA-coated samples in push-out testing in cortical sites while both surfaces supported new bone ongrowth and remodeling in cortical and cancellous sites.

**Conclusions:**

While both HA and Ti plasma provided an osteoconductive surface for bone ongrowth, the Ti plasma provided a more robust bone-implant interface that ideally would be required for load transfer and implant stability in the longer term.

## Introduction

Implant stability remains the foundation to which clinical success can be built upon for any implant in bone be it a dental implant [1, 2] or joint replacement system [3–5]. Surgical exposure and bone preparation combined with advances in manufacturing, surface technology, and geometry participate in primary stability and osseointegration during the healing process. Osseointegration, where a direct living bone-implant interface [1, 2] is achieved through bone ongrowth to a surface or ingrowth into porous domains, dictates load transfer [6], bone remodeling, and long-term fixation. Importantly, new bone formation on a surface can be encouraged by material, surface topology [7–9], porosity [3, 4, 10, 11], and chemistry [12, 13]. Improving both the rate, quantity, and quality of osseointegration has been the topic of research for decades.

What is the right surface for an implant? How rough or porous or coated does a material have to be facilitate fixation for a successful clinical outcome? The vast number of material science and manufacturing techniques available today is astounding. Choosing the “best” surface needs to consider design, manufacturing, cleaning, sterilization, mechanical properties, biocompatibility, implantation, and in vivo response. Clearly, this is a big task for all stakeholders involved in arthroplasty with the aim of achieving the best clinical outcome. The anatomic site also plays an important role in choosing an implant and a surface where osseointegration is part of the clinical paradigm [14].

Surface technologies can play important roles in encouraging interaction between the implant surface and the host bone. Hydroxyapatite (HA) coating on metal substrates has an extensive material science and preclinical foundation [7, 8, 15] as well as clinical and retrieval data [16] supporting this technology. An HA coating presents a surface that can broadly be compared to the mineral component of bone [17] that can participate in protein adsorption and cellular attachment and ideally promote osseointegration. Coating metal surfaces with titanium can also be achieved using plasma spraying techniques to provide a rough surface for bony fixation [13, 18–20]. This technology also has preclinical data [21, 22] and clinical data [18, 19, 21, 23, 24] to support its use.

This study used radiographic, mechanical testing and histology in a well-reported adult ovine model to evaluate osseointegration of two commonly used arthroplasty implant surface technologies in cortical and cancellous bone [7, 8, 12, 13, 25–30]. The null hypothesis was that there were no differences in bone fixation and mechanical properties as well as histological reaction between a hydroxyapatite-coated titanium alloy and a titanium plasma-coated titanium alloy dowel.

## Materials and methods

Titanium alloy implants in the form of cylindrical dowels (6 × 25 mm) were used in this study. The implants were vacuum plasma-sprayed Ti or grit blasted and coated with 80 μm of hydroxyapatite. One implant from each group was used for surface characterization. Stereozoom images were taken at × 1–10 magnifications using an M125C Encoded Stereo Microscope (Leica Microsystems, Wetzlar, Germany) and electron microscopy (Fig. 2) following sputter coating Emitech K575X Sputter Coater (Quorum Technologies, Lewes, UK) with FEI Nova NanoSEM 230 field-emission scanning electron microscope (FE-SEM) (Thermo Fisher Scientific, Waltham, USA). Implant surface roughness was performed with Olympus DSX510 Digital Microscope (Olympus Corp, Tokyo Japan). A portion of the HA coating was removed with a scalpel blade, and absorbance spectra generated between 400 and 4000 cm^−1^ based on 64 background subtracted scans using a Spectrum Two FT-IR Spectrometer (PerkinElmer, Waltham, USA). A hydroxyapatite standard was obtained from a local supplier (Sigma-Alderich, CAS: 1306-06-5) and analyzed under the same conditions for comparison.

All surgical procedures were performed following institutional ethical clearance on 8 adult crossbred wethers (2 years old). Animals were received from our open paddock farm and acclimatized for a minimum of 7 days prior to surgery in pairs on deep litter in climate-controlled facilities. Pre-emptive analgesic was provided using transdermal fentanyl patches 24 h before surgery and to provide smoother sedation and anesthetic induction [13]. Animals were sedated with an intramuscular (IM) injection of xylazine (0.2 mg/kg) followed by ketamine IM (6 mg/kg) 15 min later. All animals received 1 g of cephalothin (18–22 mg/kg) intravenously and 5 mL oxytetracycline (200 mg/mL) at 18 to 22 mg/kg intramuscularly. Benacillin (procaine penicillin 150 mg/mL) 1 mL/10 kg was given IM. The transdermal fentanyl patches were replaced with new ones (to provide a minimum of 72 h of postoperative analgesia [31]), and varprofen (Rimadyl 50 mg/mL) at 3 to 4 mg/kg IM given before surgery. Animals were transferred to the operating room table and anesthesia maintained using on isoflurane (1.5–3%) and oxygen (2 L/min) throughout the procedures. Animals were allowed weight bearing immediately following recovery from anesthetic. Animals were monitored and recorded daily for the first 7 days. After 7 days, they were monitored daily but only recorded weekly.

This bilateral model allows two cancellous and three bicortical implants per side. Ten dowels (five each side) were implanted using an established osseointegration model in 2-year-old adult crossbred wethers [7, 8, 12, 13, 25–30]. The sample size for this study was 8 implants per group in cortical sites and 5 implants per group in cancellous sites. This sample size has been shown to provide adequate power (beta error 10%) at alpha set to 0.05 to detect approximately a 20% difference between groups with a standard deviation of 15%. All implants were randomized in the cortical and cancellous sites. Sites were prepared with saline irrigation during drilling to minimize any thermal damage. Bicortical sites in the anteromedial aspect of the tibia were prepared with a 4.5-mm three-fluted drill (Surgibit, Orthopedic Innovations, Sydney) to create a pilot hole followed by a 6-mm-diameter drill-bit for line to line implantation of the dowels. Dowels in the cancellous sites were implanted using a gap model [26] in the cancellous bone of medial distal femoral condyles and proximal tibias. A 4.5-mm three-fluted drill pilot hole was created, over-drilled with 5.5 mm drill to create a press fit for the dowels with the site. A step drill (6, 8, and 10 mm) was used to create a 6-mm hole for the line to line, 8-mm hole for the 1-mm gap, and 10-mm hole for a 2-mm gap. The implants were inserted using an impactor into the press fit drilling scenario and centralized in the hole. The periosteum, soft tissues, and dermis were closed in layers using 3-0 and 2-0 resorbable suture, respectively.

Animals were euthanized following sedation at 4 weeks and 12 weeks. The surgical sites were examined for signs of adverse reaction or infection. The harvested bones were X-rayed in the anteroposterior and lateral views using a Faxitron (Faxitron, Wheeling, IL) and digital plates (AGFA CR MD4.0 Cassette). Radiographs in the anteroposterior and lateral views determine implant placement, adverse bony reactions, and evidence of radiographic changes at the implant bone interface. Cancellous sites were isolated using a saw, fixed in cold phosphate-buffered formalin and processing using routine polymethylmethacrylate (PMMA) embedding. The cortical sites were isolated using a saw in the axial plane. These samples were sectioned in the sagittal plane to isolate the medial and lateral specimens for push-out testing followed by PMMA hard-tissue histology. Prior to mechanical testing, the specimens were polished using a Buehler polisher perpendicular to the long axis of the implant to remove any periosteal bone overgrowth.

Implants were tested for implant-bone interface shear strength using a standard push-out test. Specimens were tested at 0.5 mm/min on a calibrated servo-hydraulic testing machine (MTS Mini Bionix®, MTS Systems Inc., Minneapolis, MN, USA). The cortical thickness was obtained from histology images and was used in shear stress calculations following formalin fixation and PMMA embedding. Peak load, stiffness, and energy to failure were determined by plotting of the load-deformation curve and calculated using a MATLAB script (MATLAB R2016a, MathWorks, Natick, MA, USA).

The shear stress was calculated according to the following relation:

$$ \sigma =\frac{Load}{\left(\frac{c_1+{c}_2}{2}\right).\pi .{d}_i} $$

where σ is the shear stress, *c*_1_ and *c*_2_ are the cortical thickness on each side of the implant in the histology section, and *d* is the implant diameter.

Formalin-fixed samples were sequentially dehydrated in increasing concentrations of ethanol before infiltration in methylmethacrylate and polymerization using established techniques. Embedded cortical and cancellous dowels were sectioned along the long axis of the implants using a Leica SP 1600 Microtome (Leica Biosystems, Nussloch, Germany). A minimum of two thin (~ 15–20 μm) sections were cut from each dowel. The sections were briefly etched in acidic ethanol (98 mL ethanol 96% and 2 mL HCl 37%) and stained with methylene blue followed by basic fuchsin. The stained slides were reviewed under low magnification to provide an overview of the section and histomorphometry. The implant-bone interface and local reactions were carefully examined at higher magnification for the presence of inflammatory cells or local particulate in the cancellous and cortical sites. The cancellous sites were also examined based on the implant conditions at press fit, line to line, and 1- and 2-mm gaps for local reactions.

PMMA images at the bone-implant interfaces were used to determine bone ongrowth and percentage of bone contact with the implant [12, 13, 26, 27]. The proximal and distal bone-implant interfaces of the cortical bone were evaluated for each slide, and a mean value based on 2 slides per site was used for statistical analysis. Similarly, the proximal and distal bone-implant interface in the cancellous sites was used to provide a mean bone ongrowth value for each implantation condition.

Mechanical and histomorphometric data was analyzed using a two-way analysis of variance (implant and time) for cortical as well as cancellous sites and post hoc testing when appropriate using SPSS (version 25, IBM, Armonk, NY, USA).

## Results

**Fig. 1:**
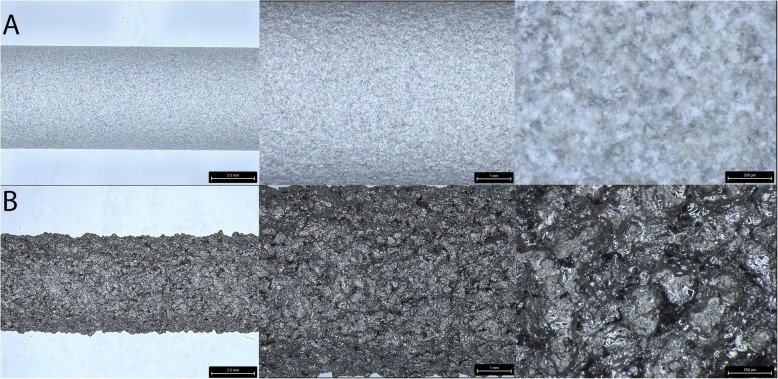
Stereozoom images of the implants examined in the study. **a** Titanium alloy dowel coated with hydroxyapatite. **b** Titanium alloy coated with plasma-sprayed titanium

**Fig. 2:**
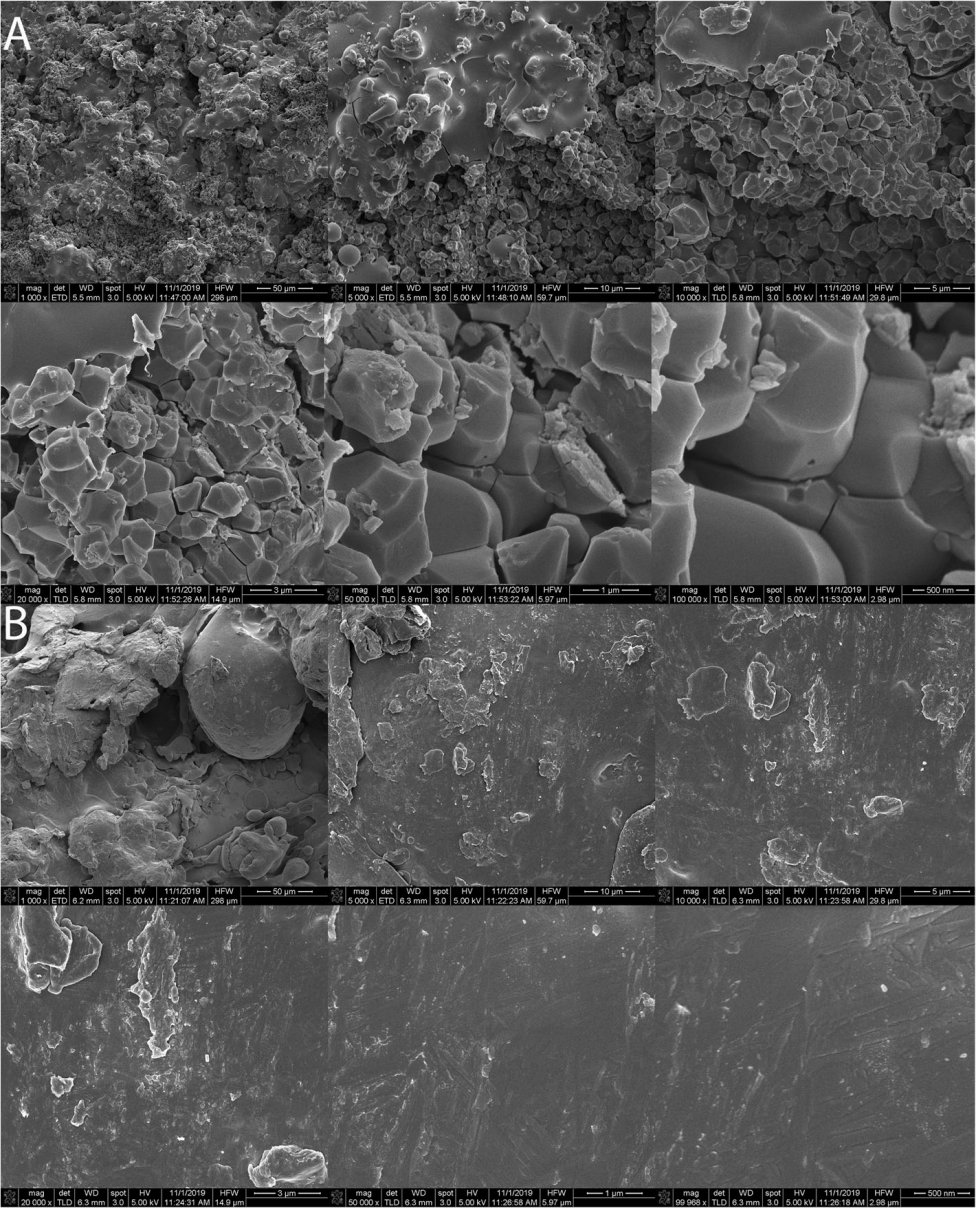
Six electron microscopy images at top row (× 1000, × 5000, × 10,000) and bottom row (× 20,000, × 50,000, and × 100,000) are presented for **a** titanium alloy dowel coated with hydroxyapatite and **b** titanium alloy coated with plasma-sprayed titanium. These images illustrate the differences in surface topography between the two implants examined in this study

**Fig. 3:**
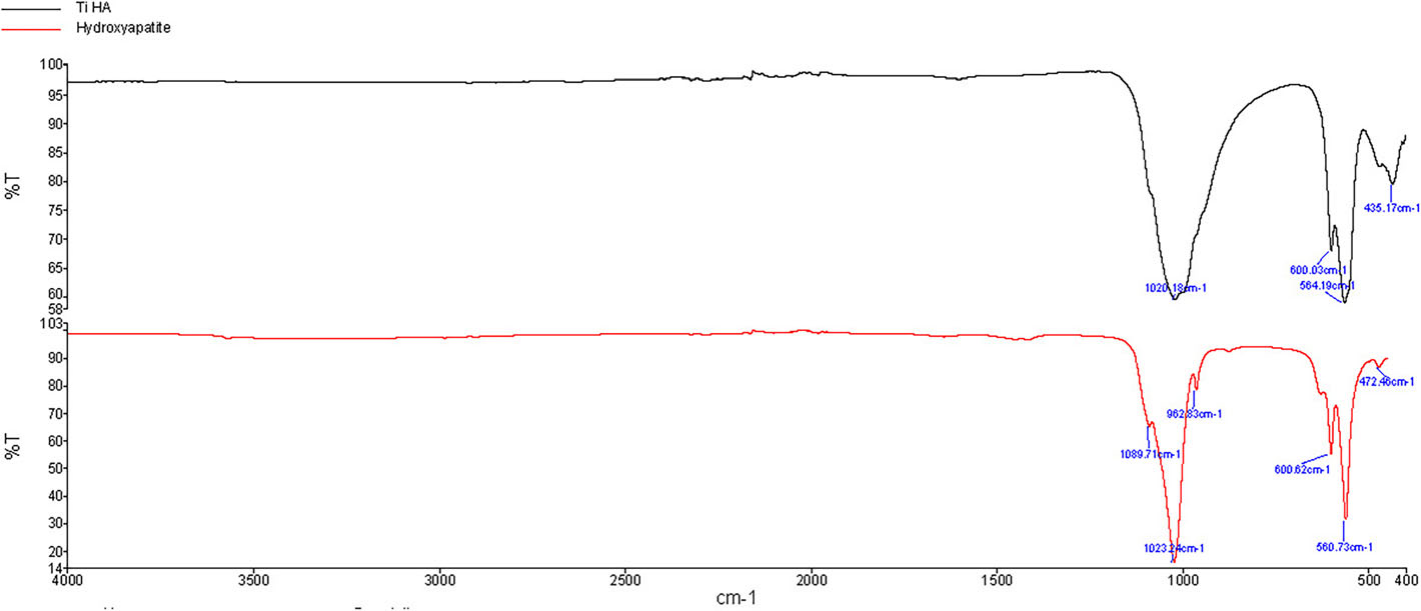
FTIR spectra for the hydroxyapatite coating which was on the Ti alloy dowel compared to a standard hydroxyapatite

The surface topology of the implants from stereozoom and electron microscopy images is presented in Figs. 1 and 2. Differences in the surface topologies can be seen for both groups at various magnifications up to × 100,000 with the globular appearance of calcium phosphate as well as the lack of distinct surface features for the Ti plasma coating beyond × 5000 magnification (Fig. 2). A uniform HA coating was present on the Ti HA samples with a surface roughness Ra of 5.557 μm. The Ra surface roughness of the Ti plasma revealed a surface was 22.906 μm. The FTIR spectra (Fig. 3) of HA standard and the HA coating collected from the implant revealed the typical spectra of calcium phosphate with major corresponding mineral peaks of phosphate (PO_4_^3−^) and carbonate (CO_3_^2−^) identified.

Surgery was completed without incident, and no adverse events were encountered during this study. All wounds were well healed, and there was no evidence of adverse reactions to either implant group in cortical or cancellous sites at both time points. Radiographs at 4 and 12 weeks revealed no evidence of adverse reactions in cortical and cancellous sites. A progression in healing in the cortical sites was noted with time for both groups as well endosteal bone formation within the marrow cavity.

Mechanical testing data is summarized in Table 1 (mean and standard deviation (SD)). The mechanical properties increased with time for both groups between 4 and 12 weeks (*P* < 0.05). No statistical differences at the *P* < 0.05 level were found at 4 weeks between groups. Statistical differences were detected at 12 weeks for force, energy, and shear stress with the Ti plasma group outperforming the Ti HA group.

**Table 1:** Mechanical data

Group	Weeks	Force (N)		Energy (Nmm)		Stiffness (N/mm)		Shear stress (MPa)	
		Mean	SD	Mean	SD	Mean	SD	Mean	SD
Ti HA	4	876.3	327.6	247.7	206.6	3879.2	1612.1	10.6	3.3
	12	1894.9	519.5	483.4	290.7	8724.0	3727.4	18.9	4.0
Ti plasma	4	1121.2	541.2	358.1	260.8	4281.1	1663.0	11.8	4.9
	12	3110.9	890.6	735.3	215.0	10,795.0	3438.5	26.5	3.4

**Fig. 4:**
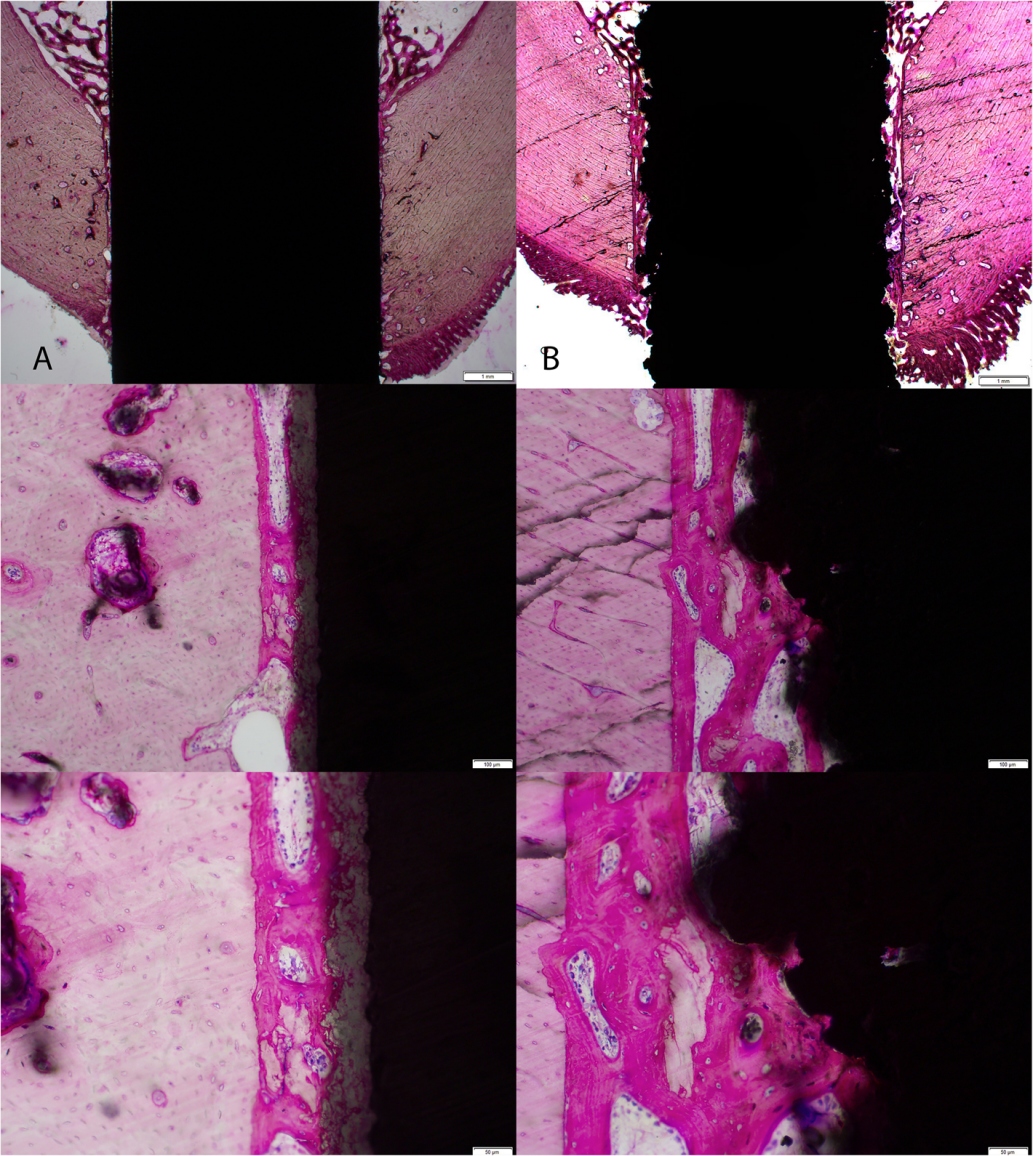
Representative histology images in the cortical sites at 4 weeks (top row × 1.25, middle × 10, bottom × 20 objectives) for the **a** titanium alloy dowel coated with hydroxyapatite and **b** titanium alloy coated with plasma-sprayed titanium. New bone ongrowth without any fibrous tissue was present for both groups

**Fig. 5:**
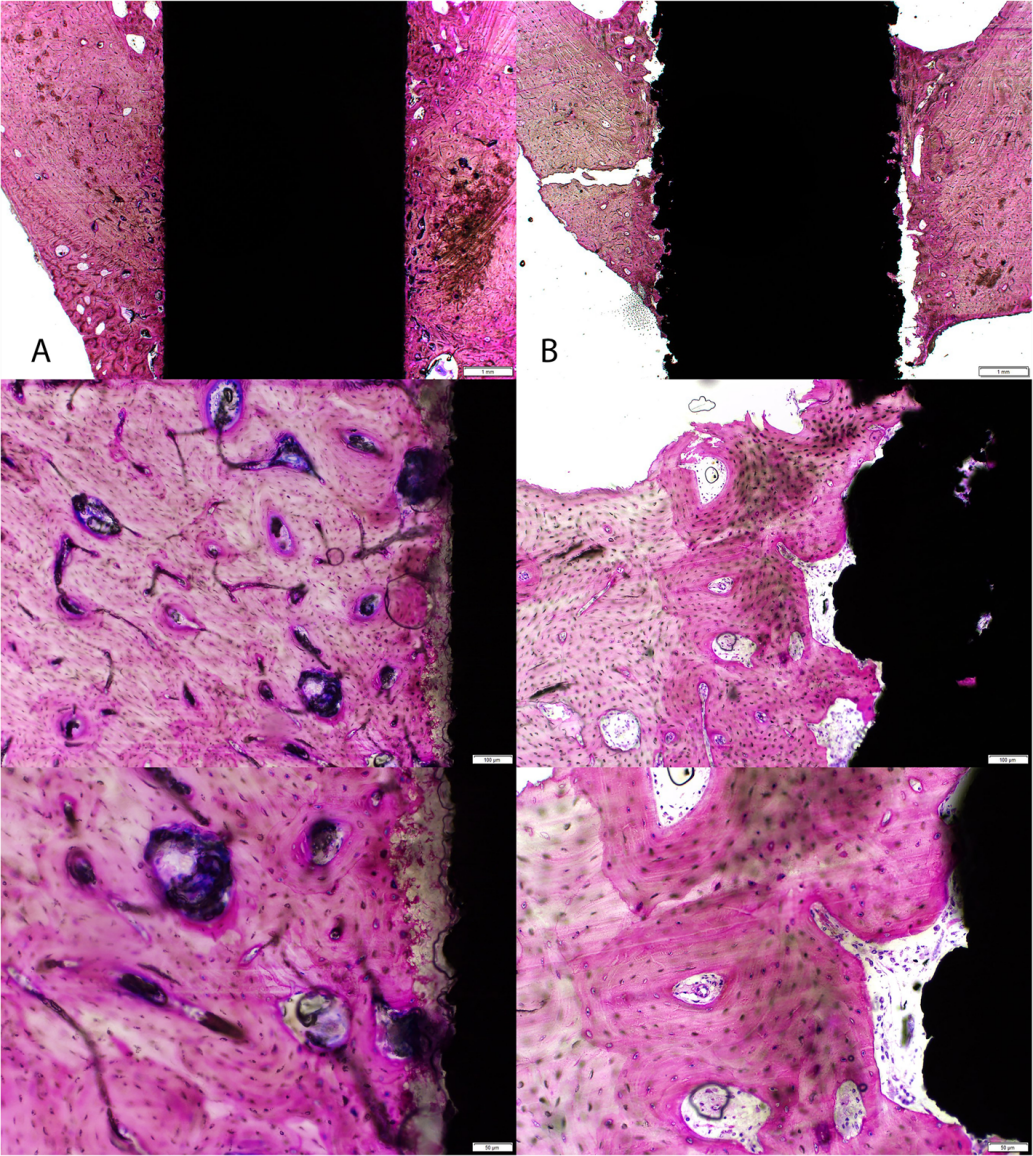
Representative histology images in the cortical sites at 12 weeks (top row × 1.25, middle × 10, bottom × 20 objectives) for the **a** titanium alloy dowel coated with hydroxyapatite and **b** titanium alloy coated with plasma-sprayed titanium. Bone remodeling without any adverse reactions was noted for both groups. The HA coating remained intact. The failure during push-out testing can be seen in the low magnification image for the Ti plasma (**b**)

**Fig. 6:**
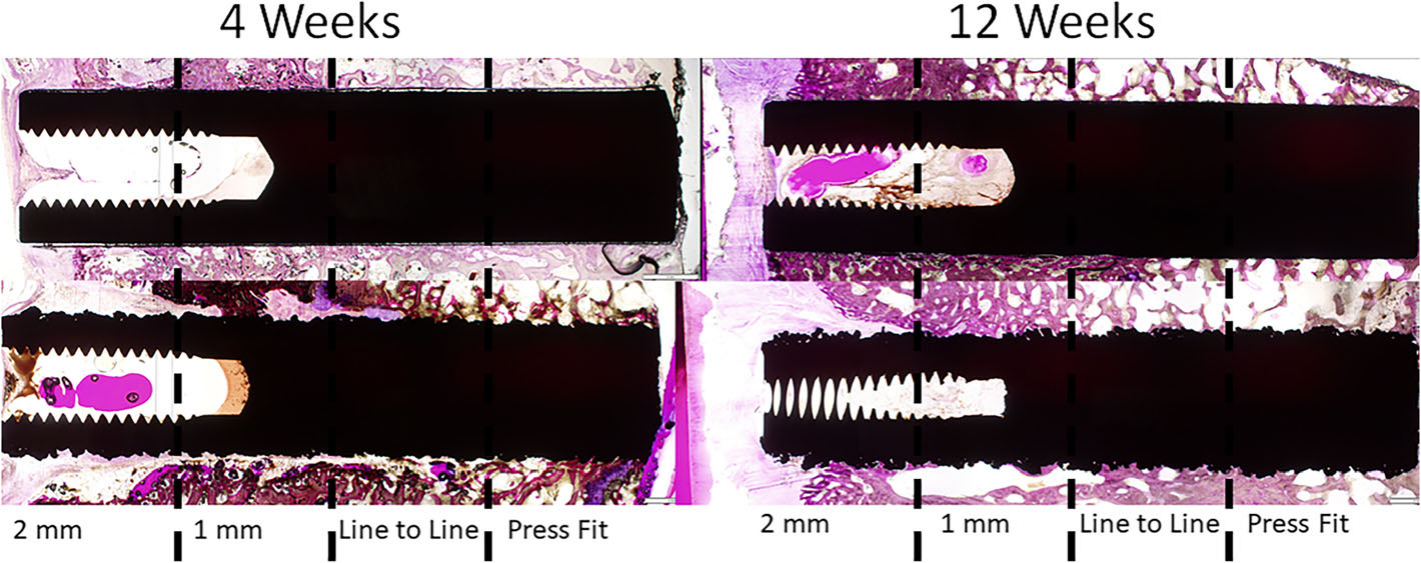
Representative histology images in the cancellous sites (2-mm and 1-mm gaps, line to line, and press fit) at 4 and 12 weeks for the **a** titanium alloy dowel coated with hydroxyapatite and **b** titanium alloy coated with plasma-sprayed titanium. Histology in the cancellous sites in the 4 implantation conditions (2-mm gap, 1-mm gap, line to line, and press fit) demonstrated the positive attributes of the HA coating in the 2- and 1-mm gap at 4 weeks with new woven bone ongrowth while the line to line to line and press fit conditions were similar. Cancellous histology at 12 weeks improved with time without the presence of any fibrous tissue and was similar for both groups

Examples of the histology in the cortical and cancellous sites at 4 and 12 weeks are presented in Figs. 4, 5, and 6. The cortical sites were implanted in a line to line manner and revealed new bone formation directly on the HA coating as well as on the Ti plasma coating without any intervening fibrous tissue interfaces at 4 and 12 weeks. Bone remodeling occurred with time for both groups in the cortical sites without any evidence of HA coating resorption between 4 and 12 weeks. Histology in the cancellous sites in the 4 implantation conditions (2-mm gap, 1-mm gap, line to line, and press fit) demonstrated the positive attributes of the HA coating in the 2- and 1-mm gap at 4 weeks with new woven bone ongrowth while the line to line and press fit conditions were similar. Cancellous histology at 12 weeks improved with time without the presence of any fibrous tissue and was similar for both groups.

**Fig. 7:**
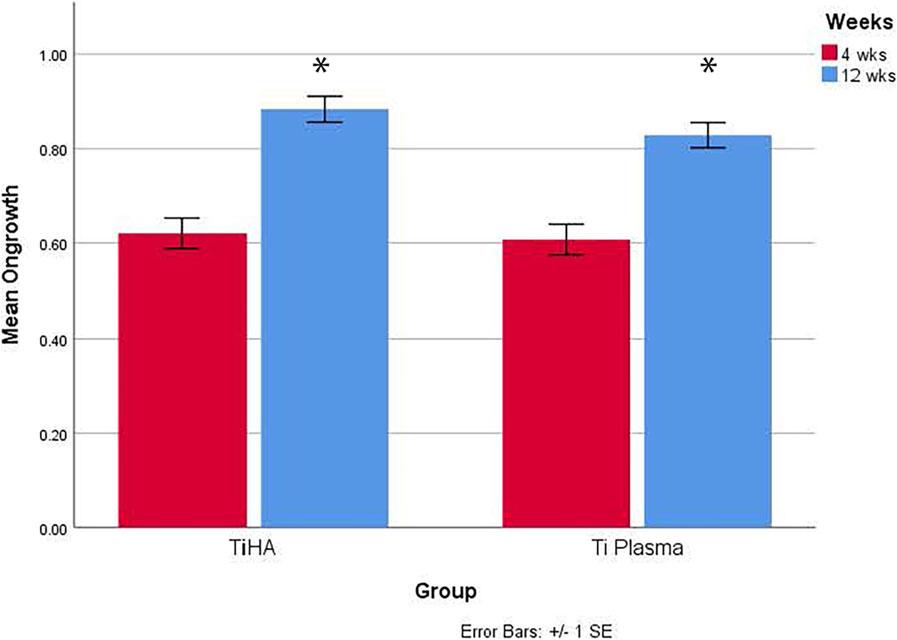
Histomorphometry for bone ongrowth in the cortical sites improved with time for both groups (*P* < 0.05) while no differences were detected between groups at 4 and 12 weeks

**Fig. 8:**
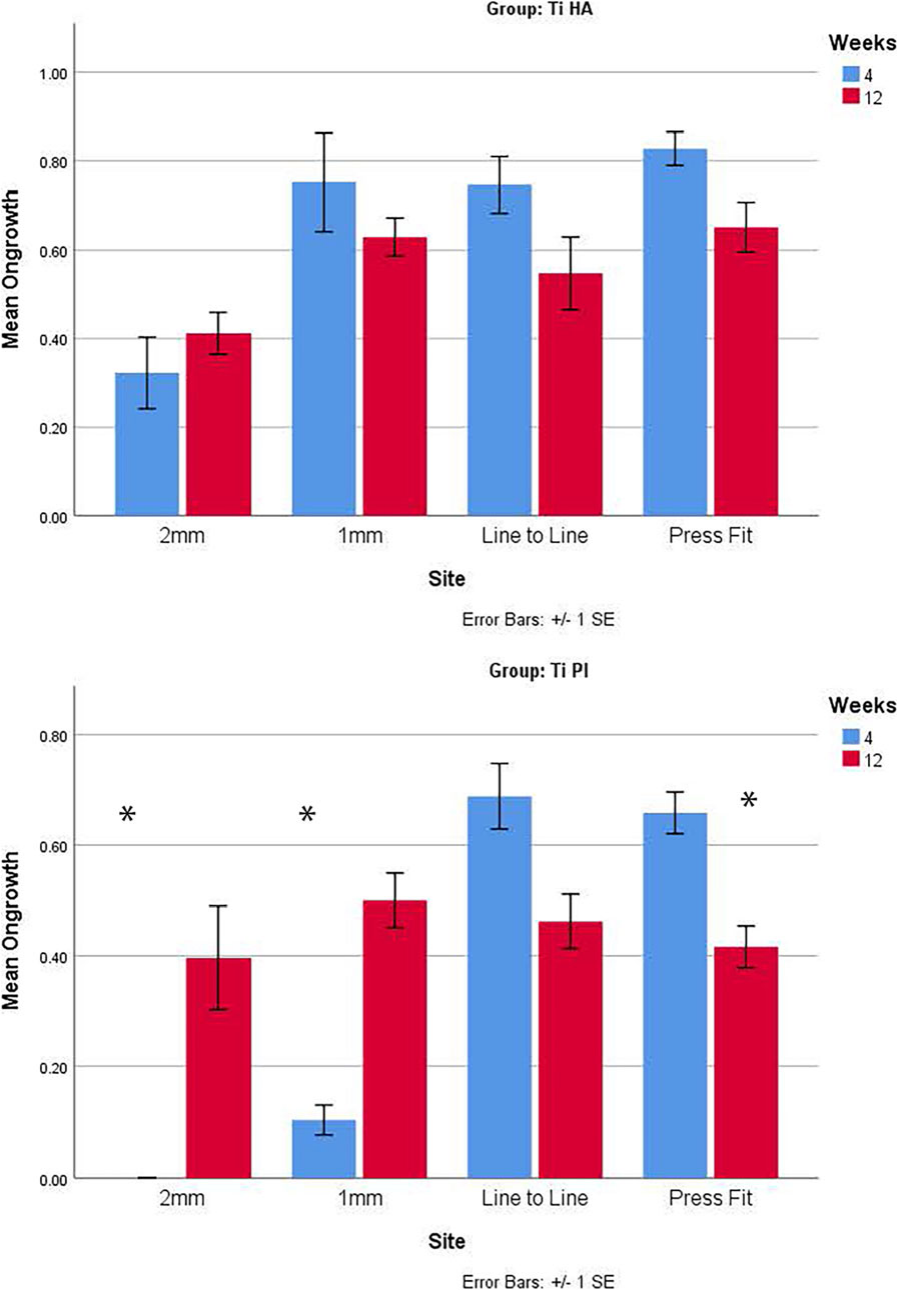
Histomorphometry in the cancellous sites at 4 weeks revealed differences between the Ti HA and Ti plasma in the 2-mm and 1-mm gap and press fit conditions (*P* < 0.05) while no differences were detected in the line to line implantation or press fit conditions. Histomorphometry in the cancellous sites at 12 weeks revealed a difference between groups in the press fit conditions (*P* < 0.05). No other differences were detected at 12 weeks

Histomorphometry for bone ongrowth in the cortical sites improved with time for both groups (Fig. 7, *P < 0.05) while no differences were detected between groups at 4 and 12 weeks (Fig. 7). Histomorphometry in the cancellous sites at 4 weeks (Fig. 8, *P < 0.05) revealed differences between the Ti HA and Ti plasma in the 2-mm and 1-mm gap and press fit conditions, with Ti HA exhibiting significantly (*P* < 0.05) more bone ongrowth than the plasma-sprayed Ti group, while no differences were detected in the line to line or press fit conditions. Histomorphometry in the cancellous sites at 12 weeks (Fig. 8) revealed a difference between groups in the press fit conditions, with Ti HA exhibiting significantly (Fig. 8, *P < 0.05) more bone ongrowth than the plasma-sprayed Ti group. No other differences were detected at 12 weeks.

## Discussion

In vitro and preclinical studies are often used as part of the development of any medical devices as a precursor to human clinical use. An understanding of how materials and their coatings respond in vivo prior to human clinical use is an important component in the development as well as evolution of medical devices. The current study compared the in vivo response of a Ti alloy implant coated with HA compared to a plasma-sprayed Ti in an established ovine model where implants were placed in the cortical as well as cancellous sites at 4 and 12 weeks. This study was limited in the lack of long-term data where bone remodeling as well as resorption of the HA coating could play a role. While both HA and Ti plasma provided an osteoconductive surface for bone ongrowth, the Ti plasma provided a more robust cortical bone-implant interface that ideally would be required for load transfer and implant stability in the longer term.

Differences in chemistry and macro-, micro-, and nano-topography were present between the Ti HA and Ti plasma implants as shown in the stereozoom, electron microscopy, surface roughness, and FTIR (Figs. 1 and 2). The bone-implant interface available for osseointegration with these features plays an important role in the in vivo response. The SEM and FTIR of the Ti HA coating were similar to osteoconductive bone graft [32, 33] and differ from osteoinductive calcium phosphate [34, 35].

Mechanical testing revealed the Ti plasma implants outperformed the Ti HA implants in the cortical sites at 4 weeks and achieved statistical significance at 12 weeks (Table 1). The fixation provided by the Ti plasma surface can be attributed to the plasma titanium coating features that present a large surface area for bony contact and some ingrowth as well as ongrowth due to the coating topography as shown in the surface roughness and imaging data. New bone formation into the peaks and valleys of the Ti plasma surfaces allows for fixation at the micro level allowing the bone-implant interfaces a greater capacity to resist shear forces. Mean bone ongrowth to the implant surfaces were similar between groups at 4 and 12 weeks and improved with time. The 4-week histology (Fig. 4) at the implant-bone interface in cortical sites demonstrated direct bone ongrowth to the surface in both groups without the presence of any intervening fibrous tissues or inflammatory cellular response. Cortical histology at 12 weeks (Fig. 5) presented an increase in bone maturity in both groups along with the presence of Haversian bone demonstrating remodeling. Bone was in direct contact with the HA coating which did not appear to have resorbed to any extent within the time frame of this experiment. The cancellous gap model was able to demonstrate the potential benefits of the HA coating where direct bone contact is limited. New bone was observed in the cancellous gap sites in the Ti HA group that could be attributed to the presence of the calcium phosphate coating and local biological benefits [8, 36]. This effect was particularly apparent at the early (4 week) time point.

Preclinical studies are models and regardless of complexity cannot replicate the human scenario where loading, articulations, wear particles, and patient-related factors including co-morbidities play a role in the overall clinical outcomes. Preclinical studies have limitations but allow for tighter control of variables such as animal age, implant geometry, and implantation conditions that can help identify the advantages as well as disadvantages of different technologies. A variety of large animal preclinical models have been used to examine the bone-implant interface with cylindrical dowels to evaluate bone ingrowth and ongrowth to different materials and surface coatings in cortical sites in sheep [7, 8, 12–14, 25–28, 37, 38] and dogs [11, 15, 39–41] as well as cancellous sites as performed in this study [12, 13, 26, 28] and intercondylar sites [37, 38, 42]. Care should always be taken when making the jump to the human clinical scenario. The current study is limited in terms of the number of time points examined. The 4-week time point represents the early aspects of healing while the 12-week time point represents an intermediate investigation. Longer time points would be potentially valuable to further differentiate implant fixation strategies in future studies. A strength of all these models is however in the detailed reporting of implant characterization, mechanical properties, and histology at the bone-implant interface that allows for important comparisons and contrasts, which is generally difficult to achieve with clinical implantations. The bicortical implant sites enable a direct measure of implant fixation and calculation of shear stress and corresponding histology at the implant-bone interface. In conclusion, both HA and Ti plasma provided an osteoconductive surface for bone ongrowth; however, the Ti plasma provided a more robust cortical bone-implant interface that ideally would be required for load transfer and implant stability in the longer term.

## Data Availability

The datasets used and/or analyzed during the current study are available from the corresponding author on reasonable request.

## Abbreviations

σ: Shear stress
*c*_1_ and *c*_2_: Cortical thickness on each side of the implant in the histology section
*d*: Implant diameter
